# A comprehensive curated resource for follicle stimulating hormone signaling

**DOI:** 10.1186/1756-0500-4-408

**Published:** 2011-10-13

**Authors:** Deepthi Telikicherla, Aditi Ambekar, Shyam Mohan Palapetta, Sutopa B Dwivedi, Rajesh Raju, Jyoti Sharma, TS Keshava Prasad, YL Ramachandra, S Sujatha Mohan, Jagadeesha Maharudraiah, Srabani Mukherjee, Akhilesh Pandey

**Affiliations:** 1 Institute of Bioinformatics, International Tech Park, Bangalore-560 066, India; 2 Department of Biotechnology, Kuvempu University, Shankaraghatta-577 451, India; 3 Department of Molecular Endocrinology, National Institute for Research in Reproductive Health (ICMR), Mumbai-400 012, India; 4 Centre of Excellence in Bioinformatics, School of Life Sciences, Pondicherry University, Puducherry-605014, India; 5 Amrita School of Biotechnology, Amrita University, Kollam-690525, India; 6 Manipal University, Madhav Nagar, Manipal-576104, India; 7 Laboratory for Immunogenomics, Research Unit for Immunoinformatics, RIKEN Research Center for Allergy and Immunology, 1-7-22 Suehiro-cho, Tsurumi-ku, Yokohama, Kanagawa 230-0045, Japan; 8 RajaRajeshwari Medical College and Hospital, Bangalore-560074, India; 9 McKusick-Nathans Institute of Genetic Medicine, Johns Hopkins University School of Medicine, Baltimore, MD 21205, USA; 10 Department of Biological Chemistry, Johns Hopkins University School of Medicine, Baltimore, MD 21205, USA; 11 Department of Pathology, Johns Hopkins University School of Medicine, Baltimore, MD 21205, USA; 12 Department of Oncology, Johns Hopkins University School of Medicine, Baltimore, MD 21205, USA

## Abstract

**Background:**

Follicle stimulating hormone (FSH) is an important hormone responsible for growth, maturation and function of the human reproductive system. FSH regulates the synthesis of steroid hormones such as estrogen and progesterone, proliferation and maturation of follicles in the ovary and spermatogenesis in the testes. FSH is a glycoprotein heterodimer that binds and acts through the FSH receptor, a G-protein coupled receptor. Although online pathway repositories provide information about G-protein coupled receptor mediated signal transduction, the signaling events initiated specifically by FSH are not cataloged in any public database in a detailed fashion.

**Findings:**

We performed comprehensive curation of the published literature to identify the components of FSH signaling pathway and the molecular interactions that occur upon FSH receptor activation. Our effort yielded 64 reactions comprising 35 enzyme-substrate reactions, 11 molecular association events, 11 activation events and 7 protein translocation events that occur in response to FSH receptor activation. We also cataloged 265 genes, which were differentially expressed upon FSH stimulation in normal human reproductive tissues.

**Conclusions:**

We anticipate that the information provided in this resource will provide better insights into the physiological role of FSH in reproductive biology, its signaling mediators and aid in further research in this area. The curated FSH pathway data is freely available through NetPath (http://www.netpath.org), a pathway resource developed previously by our group.

## Background

Follicle stimulating hormone (FSH) is a glycoprotein hormone secreted by cells called gonadotrophs in the anterior pituitary gland. The major function of FSH is to promote and sustain the ovarian follicular growth in female [1] and spermatogenesis in male [2]. FSH stimulates the synthesis of its own receptor on the granulosa cells of the ovary [3] and in Sertoli cells [4] of the testes. Treatment with FSH also results in the expression of the luteinizing hormone (LH) receptor on granulosa cells [5]. FSH secretion is under the control of pulsatile GnRH release from hypothalamus. The production of estrogen, progesterone and inhibin from ovarian cells is, in turn, controlled by FSH. Estrogen and inhibin regulate FSH secretion through negative feedback on the pituitary [6]. All these dynamic changes in the hypothalamo-pituitary-ovarian axis are required for selection of the dominant follicle, ovulation and thus the menstrual cycle.

FSH is a member of glycoprotein hormone family, which also includes LH, human chorionic gonadotropin (hCG) and the thyroid stimulating hormone (TSH). These hormones are heterodimeric proteins comprised of a common α subunit and a unique β subunit that confers the biological specificity to each hormone. The receptors of these hormones belong to the G-protein coupled receptor (GPCR) superfamily, members of which contain seven transmembrane alpha helical domains [7].

Binding of FSH to its cognate receptor, FSH receptor (FSHR), triggers rapid activation of multiple signaling cascades. FSH action is primarily mediated via activation of the receptor associated heterodimeric G-proteins, which results in stimulation of adenylate cyclase activity and production of cyclic AMP (cAMP) [8]. Consequently, the cAMP-dependent protein kinase (PKA) is activated which in turn leads to phosphorylation of several transcription factors including cAMP responsive element binding protein (CREB), as well as chromatin remodeling through histone H3 modifications [9,10]. Cyclic AMP/PKA also enhances the activity of p38 mitogen-activated protein kinase (p38MAPK), extracellular signal regulated-kinase (ERK) and phosphatidylinositol-3-kinase (PI3K), which can also be activated by PKA-independent manner [11]. These activated kinases further activate specific transcription factors, which regulate expression of FSH-target genes including aromatase [12], vascular endothelial growth factor VEGF [13], serum- and glucocorticoid-inducible kinase (SGK) [14] and steroidogenic acute regulatory protein (STAR) [15]. Early response genes induced by FSH include cyclin D2, the regulatory subunit of protein kinase A (RII-β) and SGK and the late response genes include cycloxygenase-2 (COX-2) and LH receptor (LHR) [16]. In addition, FSH activates the transcription factor hypoxia-inducible factor-1 (HIF-1) downstream of the PI3-kinase/AKT/Rheb/mTOR axis [17]. Apart from cAMP, FSH increases intracellular concentrations of other second messengers including calcium and inositol 1, 4, 5-triphosphate (IP3) [18,19].

Although FSH signaling has been relatively well studied, there is a dearth of resources, which provide comprehensive information about the molecules involved in the FSH signaling pathway, the transcription factors activated by FSH and the genes whose expression is eventually regulated. Several online pathway databases including The Database of Cell Signaling (http://stke.sciencemag.org/cm/), KEGG Pathway Database (http://www.genome.jp/kegg/pathway.html), Pathway Interaction Database of the National Cancer Institute (http://pid.nci.nih.gov/) and INOH Pathway Database (http://www.inoh.org/) contain generic information about GPCR signaling. None of these resources provide information specific to FSH-induced signaling. Because of the importance of this pathway in human physiology and disease, we undertook a systematic effort to gather and curate the relevant information available in published literature to create a public resource of the signaling events triggered by FSH.

We have previously developed NetPath [20], a public resource of signaling pathways, with the initial set of annotated pathways focused on those important in immunology and cancer biology. As a next step, we set out to extend this list and generate additional signaling pathways that are relevant to a variety of human physiological processes. Here, we report the development of a pathway map consisting of individual biochemical events that are triggered by the activation of FSH receptor by FSH.

### Pathway reactions and criteria for curation

FSH signaling pathway reactions have been curated in three categories-molecular associations, enzyme-substrate reactions and protein transport reactions. The curation of FSH pathway reactions were based on the following criteria: (i) reactions should be stimulated by FSH/FSHR system *in vivo *and compared to an unstimulated state; (ii) proteins involved in reactions should be from human system, however other mammalian proteins were annotated if they were not reported from human system; and, (iii) only experiments carried out in cell lines of mammalian origin were considered.

The molecular association reactions include both direct (binary association) and complex (multimeric association) protein-protein interactions stimulated by activated FSHR. The enzyme-substrate reactions induced by FSH were curated as direct (where the immediate upstream enzyme responsible is known) or induced (where the immediate upstream enzyme is not known) reactions. The protein sub-cellular translocation events induced by FSH were curated as transport reactions. In many studies, the authors investigate only the activation status of molecules under stimulation with the ligand. These reactions which cannot be categorized as either molecular associations or enzyme-substrate reactions were documented under a separate category called activation/inhibition reactions. Reactions in the FSH pathway were curated from literature dealing with studies carried out in mammalian host systems with the reactions reported in humans preferred over mouse, rat, bovine or other mammals.

### Generation of a curated resource of FSH signaling

**Figure 1:**
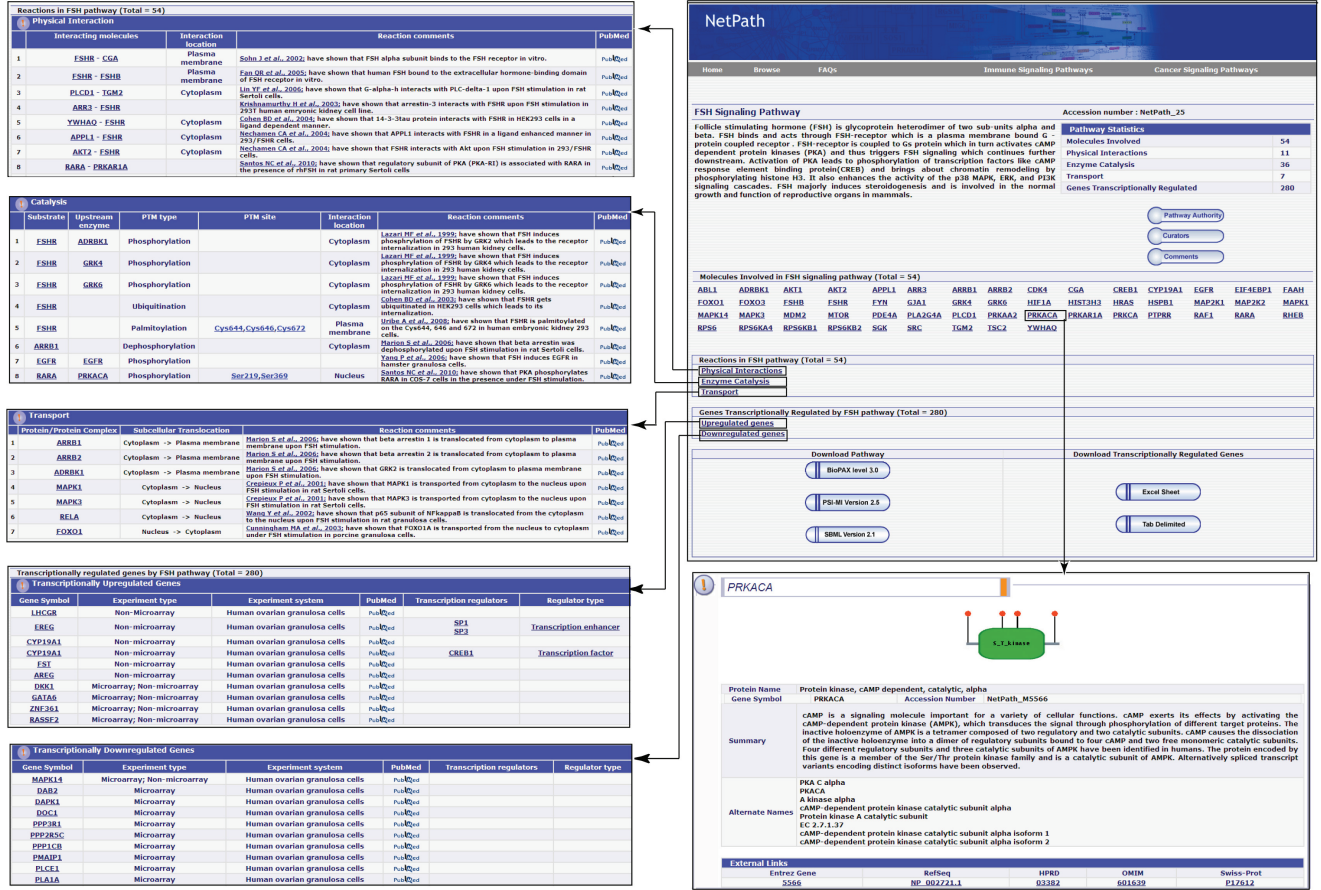
**An overview of the FSH pathway page in NetPath**. The FSH pathway page in NetPath displays the statistics of data in the FSH pathway reactions including the number of proteins, protein-protein interactions, enzyme-substrate reactions, translocation events and differentially regulated genes in normal/primary human cells. The complete list of these reactions is accessible through the specific links provided for each reaction. Brief comments are provided for reactions curated under reactions such as PPI, enzyme-substrate reactions and transport. Each of these molecules is linked to NetPath molecule page which is further linked to Entrez gene, HPRD [30], OMIM [31] and Swiss-Prot [32] identifiers. A list of curators/reviewers is provided in the FSH pathway page with the details of the Pathway Authority. A 'comments' tab is provided in the pathway page to invite queries and suggestions from the community as a means to keep the pathway updated and as error-free as possible.

The articles relevant to FSH induced signal transduction events were retrieved from PubMed. From these research articles, we documented 11 molecular association events, 35 enzyme-substrate reactions, 11 activation events and 7 protein translocation events that occur upon FSH stimulation. The site of post-translational modifications were also documented and mapped to RefSeq whenever this information was available. FSH pathway reactions are available in NetPath at (http://www.netpath.org/pathways?path_id=NetPath_25) and will be periodically updated. Figure 1 provides an overview of FSH pathway page in NetPath.

### Visualization of FSH pathway

**Figure 2:**
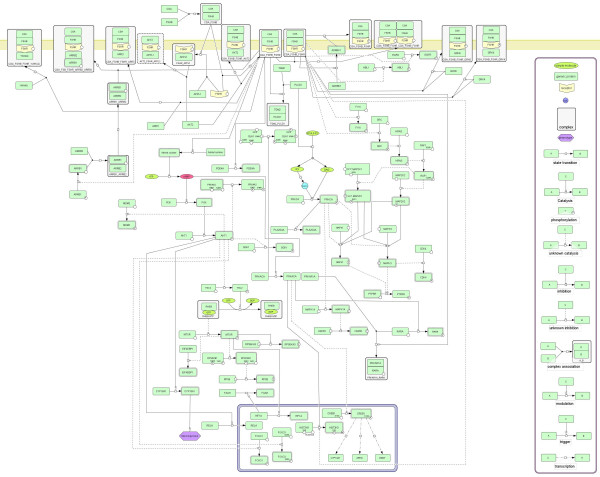
**FSH pathway reaction map generated using CellDesigner**. The reactions in the pathway resource were used to obtain a descriptive network of reactions using CellDesigner. The legend for the pathway map is provided in the box to the right. The reactions are broadly arranged according to the cellular component they occur in and are depicted here on the plasma membrane (yellow thick line at the top) and the nucleus (the rectangle with the blue border at the bottom) and the cytosol (the space between the yellow line and the blue rectangle). The small molecules such as cyclic AMP (cAMP), calcium (Ca++), inositol 3-phosphate (IP3) and diacylglycerol (DAG) which are important mediators of FSH signaling are also represented in the map.

There have been efforts by other groups to catalog and represent reactions of signal transduction pathways [21-23]. They have used Systems Biology Graphical Notation (SBGN) for representation of the pathways [24]. Therefore, we used CellDesigner Version 4.1 (http://www.celldesigner.org/) to generate the pathway map in the SBGN format (Figure 2). A high resolution image of the map is provided as Additional File 1. Apart from the protein components involved, small molecules like cAMP, IP3, DAG and Ca^++ ^which play a major role in FSH signaling are also represented in the map. Many of the reactions in the current pathway map are derived from a single experiment and may be less reliable. Thus, we have also generated a slim version of the FSH pathway map by applying more stringent criteria (http://www.netpath.org/netslim/fsh_pathway.html). A detailed description of the NetSlim criteria is available at the same portal.

### Genes regulated by FSH

Apart from the molecular association, catalysis and translocation events, we included information regarding the genes that are differentially regulated by activated FSHR in normal human reproductive tissues. We documented 265 such genes whose expression levels changed in response to FSH administration. Out of these, 166 genes were upregulated and 99 were downregulated following FSH induction of human ovarian granulosa cells. Five of the eight articles which were curated were high-throughput mRNA expression profiling studies. The differential expression of some genes was also validated using RT-PCR in two of these studies. We included data only from studies carried out in normal human cells/tissues. A minimum fold change value of 2 and a p-value < 0.05 were taken as arbitrary cutoffs for inclusion into the resource. Data was not annotated from studies carried out in any disease setting. We also excluded studies where other hormones such as LH or hCG were used along with FSH for granulosa cell stimulation as there was ambiguity about the genes regulated by individual hormones. We have also documented several instances where specific transcription factors induced by FSH were shown to control the expression of differentially regulated genes.

### Availability and data formats

The FSH pathway data is compatible with various standard data exchange formats including Proteomics Standards Initiative Molecular Interaction XML format (PSI MI) [25], a standard format for data representation in proteomics to facilitate data comparison, exchange and verification; BioPAX (http://www.biopax.org/) [26], a standard language that enables integration, exchange, visualization and analysis of biological pathway data and the Systems Biology Markup Language (SBML) (http://sbml.org/), a computer-readable format for representing models of biological processes [27]. This allows interoperability with other data analysis software tools such as Cytoscape, Visualization and layout services for BioPAX pathway models (VISIBIOweb) [28] or Chisio BioPAX Editor (ChiBE) [29]. The data for this pathway is freely downloadable from http://www.netpath.org/pathways?path_id=NetPath_25. The SBML format of the map given in Figure 2 is provided as Additional File 2.

### Review by Pathway Authority

Although the information in this resource is curated from published literature in accordance with our curation strategy and had been subjected to various levels of internal review, we consider it necessary that an expert in this area is involved to ensure its accuracy and comprehensiveness. For this pathway, the data was reviewed by S.M, who is one of the co-authors, and is the designated Pathway Authority (http://www.netpath.org/pathway_authority?path_id=NetPath_25). We hope to continue this model for curation of pathways in the future where we involve experts on individual pathways as Pathway Authorities and include them as co-authors on descriptions of the corresponding pathways.

## Conclusions

Studies of molecular events that result from activation of various receptors by their ligands are key to understanding various biological processes. Availability of such information serves as an invaluable tool for analyzing various kinds of high-throughput data obtained from gene/protein arrays as well as proteomic experiments. These analyses may include overlaying the high-throughput data onto known pathway reactions as well as perturbation analyses. We here describe the generation of a pathway map of the signaling events that are mediated by activated FSHR, through a systematic and detailed curation of the relevant published literature. This is aimed at providing a global and comprehensive view of the intracellular signaling events as well as the biological processes regulated by FSH. This would also enable researchers to perform various pathway as well as systems biology type of analyses on high-throughput data.

## Competing interests

The authors declare that they have no competing interests.

## Authors' contributions

AP designed the study. DT collected and annotated information into the resource. AA, SMP, SBD, RR assisted with the annotation. JS handled the computational aspects of the study. SM scrutinized and reviewed the pathway information as the Pathway Authority. TSKP, JM, YLR, SSM and RR also helped in reviewing the pathway reactions. DT prepared the manuscript. SM, TSKP, SMP, RR and AP edited the manuscript. All authors read and approved the final manuscript.

## Supplementary Material

Additional file 1 **FSH pathway map**. A high resolution image of FSH pathway map drawn using CellDesigner software. Click here for file

Additional file 2 **SBML file of FSH pathway**. The SBML file generated by exporting the FSH pathway map that was drawn using CellDesigner software. Click here for file
