# Supplementary material for: A comprehensive curated resource for follicle stimulating hormone signaling
Source: BMC Res Notes. 2011 Oct 13;4:408. doi: 10.1186/1756-0500-4-408 (PMC3204250; doi:10.1186/1756-0500-4-408)
Supplement: Additional file 1 — FSH pathway map. A high resolution image of FSH pathway map drawn using CellDesigner software. [file 1756-0500-4-408-S1.PDF]

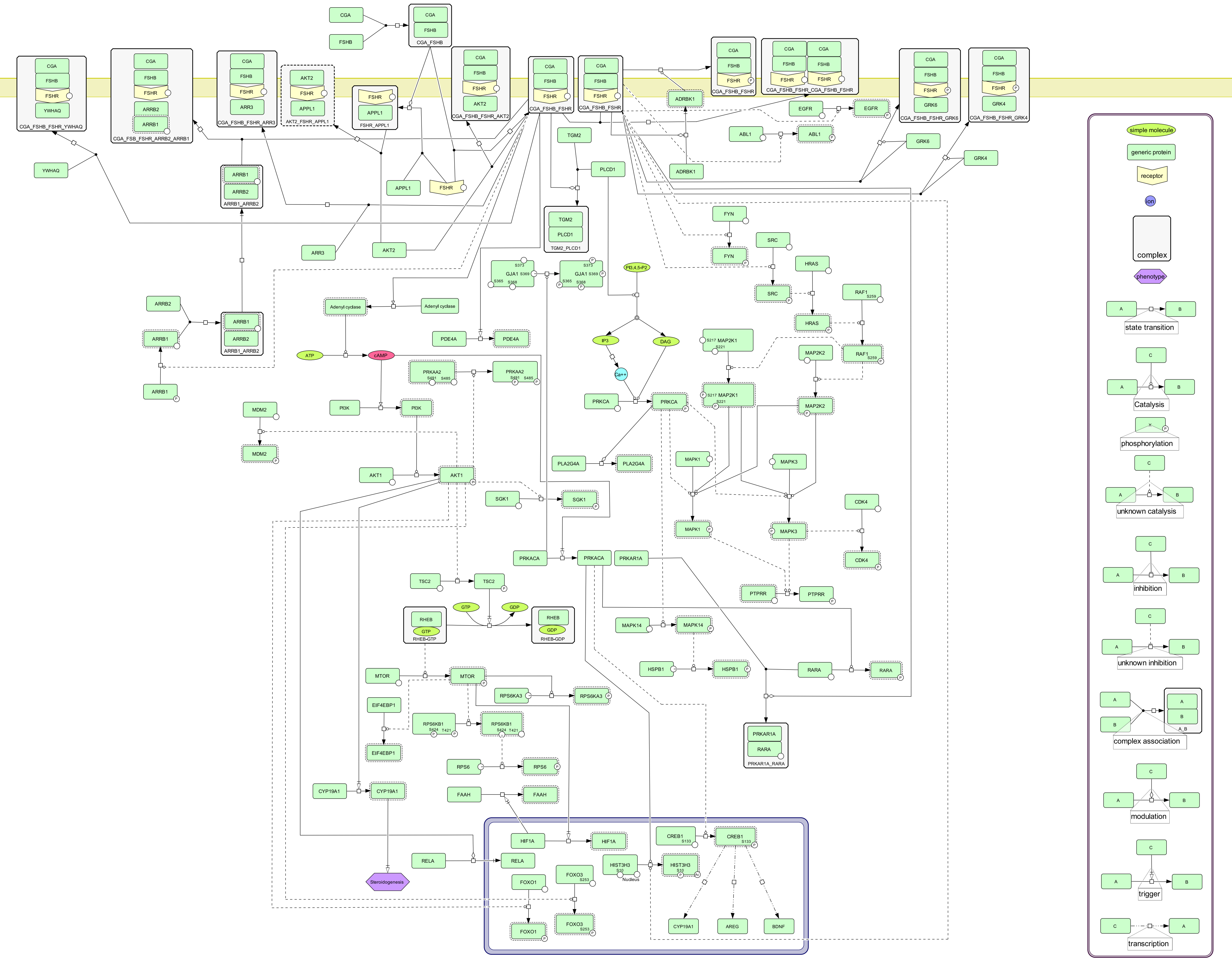

CGA
FSHB
CGA_FSHB
FSHR
YWHAQ
CGA_FSHB_FSHR_YWHAQ
ARRB2
ARRB1
CGA_FSB_FSHR_ARRB2_ARRB1
ARR3
CGA_FSHB_FSHR_ARR3
AKT2
APPL1
AKT2_FSHR_APPL1
FSHR_APPL1
CGA_FSHB_FSHR_AKT2
CGA_FSHB_FSHR
ADRBK1
CGA_FSHB_FSHR_CGA_FSHB_FSHR
EGFR
ABL1
GRK6
CGA_FSHB_FSHR_GRK6
GRK4
CGA_FSHB_FSHR_GRK4
ARRB1_ARRB2
TGM2
PLCD1
TGM2_PLCD1
FYN
SRC
HRAS
RAF1
S259
GJA1
S373
S369
S365
S368
PI3,4,5-P2
IP3
DAG
Ca++
Adenyl cyclase
ATP
cAMP
PDE4A
PRKAA2
S491
S485
PI3K
PRKCA
MAP2K1
S217
S221
MAP2K2
MDM2
AKT1
PLA2G4A
MAPK1
MAPK3
SGK1
CDK4
PRKACA
PRKAR1A
TSC2
PTPRR
RHEB
GTP
GDP
RHEB-GTP
RHEB-GDP
MAPK14
HSPB1
MTOR
RPS6KA3
RARA
EIF4EBP1
RPS6KB1
S424
T421
PRKAR1A_RARA
RPS6
FAAH
CYP19A1
HIF1A
CREB1
S133
RELA
HIST3H3
S10
Nucleus
FOXO3
S253
FOXO1
Steroidogenesis
AREG
BDNF
simple molecule
generic protein
receptor
ion
complex
phenotype
state transition
Catalysis
phosphorylation
unknown catalysis
inhibition
unknown inhibition
complex association
A_B
modulation
trigger
transcription
